# Development of a fully automated low‐pressure [^11^C]CO carbonylation apparatus

**DOI:** 10.1002/jlcr.3866

**Published:** 2020-08-27

**Authors:** Mélodie Ferrat, Youssef El Khoury, Peter Larsen, Kenneth Dahl, Christer Halldin, Magnus Schou

**Affiliations:** ^1^ Department of Clinical Neuroscience, Center for Psychiatry Research Karolinska Institutet and Stockholm County Council Stockholm Sweden; ^2^ Scansys ApS Copenhagen Denmark; ^3^ Radiopharmacy Karolinska University Hospital Stockholm Sweden; ^4^ AstraZeneca PET Science Centre, Precision Medicine, Oncology R&D, AstraZeneca Karolinska Institutet Stockholm Sweden

**Keywords:** carbon‐11, carbonylation, GMP, radiochemistry, radiochemistry module

## Abstract

[^11^C]carbon monoxide ([^11^C]CO) is a versatile synthon for radiolabeling of drug‐like molecules for imaging studies with positron emission tomography (PET). We here report the development of a novel, user‐friendly, fully automated, and good manufacturing practice (GMP) compliant low‐pressure synthesis module for ^11^C‐carbonylation reactions using [^11^C]CO. In this synthesis module, [^11^C]CO was reliably prepared from cyclotron‐produced [^11^C]carbon dioxide ([^11^C]CO_2_) by reduction over heated molybdenum and delivered to the reaction vessel within 7 min after end of bombardment, with an overall radiochemical yield (RCY) of 71%. [^11^C]AZ13198083, a histamine type‐3 receptor ligand, was used as a model compound to assess the functionality of the radiochemistry module. At full batch production conditions (55 μA, 30 min), our newly developed low‐pressure ^11^C‐carbonylation apparatus enabled us to prepare [^11^C]AZ13198083 in an isolated radioactivity of 8540 ± 1400 MBq (*n* = 3). The radiochemical purity of each of the final formulated batches exceeded 99%, and all other quality control tests results conformed with specifications typically set for carbon‐11 labeled radiopharmaceuticals. In conclusion, this novel radiochemistry system offers a convenient GMP compliant production drugs and radioligands for imaging studies in human subjects.

## Graphical abstract

Automated ^11^C‐carbonylation module allowed for GMP production of AZ13198083 in high radiochemical yield and purity.

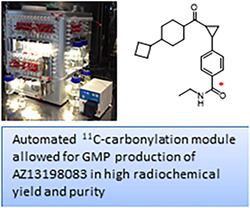

## 1 INTRODUCTION

Although [^11^C]carbon monoxide ([^11^C]CO) has been applied in radiochemistry for several decades, it has not yet gained ground as a mainstream labeling agent in the positron emission tomography (PET) radiochemistry community.^1^, ^2^ Nevertheless, many important methodological developments have been made that have facilitated its use as a synthon in PET radiochemistry,^3^, ^4^, ^5^, ^6^ in particular in transition metal‐mediated ^11^C‐carbonylation reactions.^7^, ^8^, ^9^, ^10^, ^11^, ^12^ The most explored methodology to date is probably the high‐pressure autoclave method,^13^ but due to its technically sophisticated setup, the focus has recently been directed to different ways of performing ^11^C‐carbonylation reactions at ambient pressure.^14^, ^15^, ^16^ The aim of the current work was to develop a radiochemistry apparatus that would enable application of the recently developed protocol for low‐pressure ^11^C‐carbonylation reported in our laboratory by Dahl et al.^15^

Such an apparatus would be the first commercially available apparatus for this purpose and potentially play an important role in further spreading the use of [^11^C]CO as a synthon in the PET radiochemistry community.

## 2 RESULTS AND DISCUSSION

**FIGURE 1:**
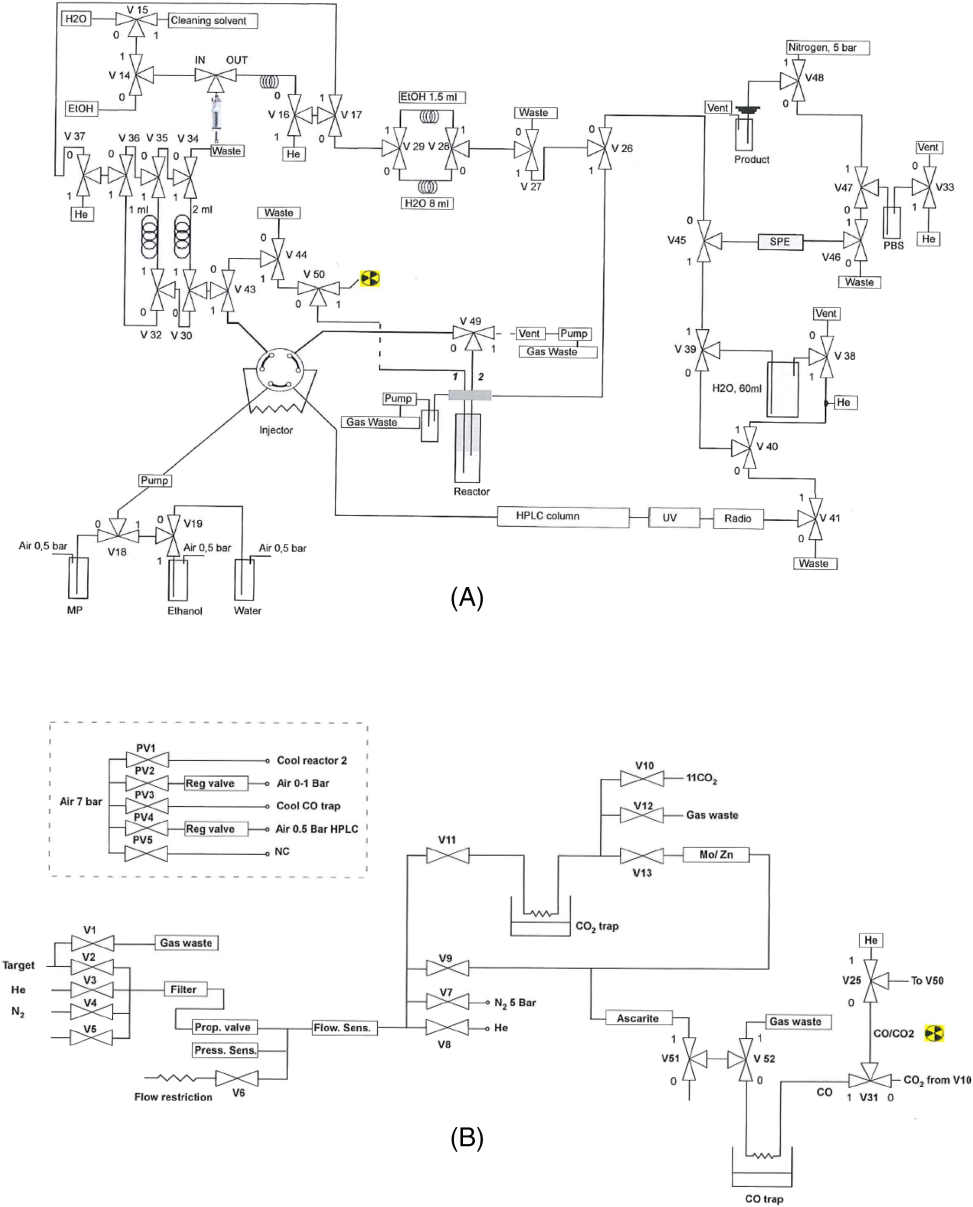
A, Flowchart of the “wet” part of the automated system for [^11^C]carbon monoxide ([^11^C]CO) radiochemistry. Cleaning solvent—tetrahydrofuran (THF) or acetone. B, Flowchart of the “dry” part of the automated system for [^11^C]CO radiochemistry

The [^11^C]CO radiochemistry apparatus was designed to handle all parts of a radiopharmaceutical preparation in an integrated, automated, and good manufacturing practice (GMP) compliant fashion. The system was in turn divided into two main subsystems. First, the radioactive gas handling system, in which [^11^C]carbon dioxide ([^11^C]CO_2_) was trapped from the target gas, reduced online to [^11^C]CO, and subsequently concentrated and transferred into the glass vial for the radiolabeling reaction. The second part was the purification and formulation subsystem, in which the crude product was first purified using high‐performance liquid chromatography (HPLC), isolated from the solvents in the mobile phase using solid phase extraction (SPE) and finally formulation in a suitable vehicle for intravenous injection and sterile filtration. The radiochemistry apparatus was controlled and monitored with Labview‐based software. A flowchart of the radiochemistry system is shown in Figure 1A,B.

## 3 RADIOACTIVE GAS HANDLING SYSTEM

The initial focus was to develop a reliable process for the production gaseous [^11^C]CO and its delivery into a sealed 3.5‐ml glass reaction vessel. Thus, a series of short [^11^C]CO_2_ productions were conducted using set irradiation conditions, 35 μA for 1 min, to calculate the efficiency of each step of the radioactive gas handling system. The starting radioactivity for the process was estimated from three consecutive [^11^C]CO_2_ productions that were trapped on an ascarite trap installed at the radioactive gas inlet to the radiochemistry apparatus (Table 1, entry 1). [^11^C]CO_2_ was produced in a nitrogen‐oxygen (1%) gas target, passed through a phosphorous pentoxide trap, and trapped in a stainless steel tube (1/4″) filled with Porapak Q (80–100 mesh) that was immersed in liquid nitrogen. The trap was flushed with He, first at −196°C and then while thawing under a stream of compressed air, with the intent to purify [^11^C]CO_2_ from residual target gas and other chemical impurities. As expected, [^11^C]CO_2_ could be efficiently trapped and released with this experimental setup (Table 1, entry 2), which has the advantage of a longer lifetime and the potential to provide a higher molar activity (A_m_) than that obtained with molecular sieves, which continually traps CO_2_ from the atmosphere. On the other hand, a molecular sieve setup does not require any liquid nitrogen and may be more efficient in removing chemical impurities from the produced [^11^C]CO_2_. No additional traps were used to purify [^11^C]CO_2_ before it was introduced into the next step of the production sequence.

**TABLE 1:** Process parameters measured during productions with low radioactivity

Entry	Measured radioactivity (MBq)^a^	Time (min)^b^	Yield (uncorrected, %)^c^	Yield (corrected, %)^d^
Starting radioactivity	3108 ± 250	6.5	100	100
Reactor	1618 ± 160	9	50	71

^a^ Average of three runs.

^b^ Time after end of bombardment until measurement.

^c^ Yield calculated without taking the half‐life into account.

^d^ Yield calculated with taking the half‐life into account.

[^11^C]CO was prepared by online reduction of [^11^C]CO_2_ in a stream of helium through a heated column filled with Molybdenum powder. Though the conversion of [^11^C]CO_2_ to [^11^C]CO was reproducible under these conditions, it is worth noting that a pressure increase was observed following repeated use of the column, which necessitated its replacement after approximately 50 runs. It is possible that an additional purification of the [^11^C]CO_2_ could increase the lifetime of this column, but no additional efforts were put into this given the relative low replacement frequency of the Mo column. The produced [^11^C]CO was purified over an ascarite trap and finally concentrated from the gas stream in a stainless‐steel tube (1/16″) filled with silica (~110 mg) before being released into a sealed glass vial containing the ^11^C‐aminocarbonylation reaction mixture.

A capillary tubing was used as transfer line for the concentrated [^11^C]CO to minimize the dead volume between the silica trap and the reactor. Although the pressure was not measured in the reaction vessel, it is likely governed by the vapor pressure of tetrahydrofuran (THF) at the reaction temperature during the ^11^C‐aminocarbonylation reaction. In the current study, in which the reaction temperature was 100°C, the pressure would thus be expected to reach approximately 2.8 bar. It is important to note that the reaction vessel is not vented until the end of the ^11^C‐aminocarbonylation reaction, which introduces a risk of radioactivity leakage in case the septum of the vessel leaks. However, in our experience, this risk can be efficiently managed by a simple leak check of the reaction vessel prior to its installation into the radiochemistry apparatus. We also considered introducing the [^11^C]CO into a vented vial to simplify the [^11^C]CO transfer, because a single pass introduction of [^11^C]CO can proceed with as high trapping efficiency as 95%.^15^ However, the trapping efficiency depends on several factors, and not all combinations of substrate, catalyst, and ligand can proceed with such efficiency. It was thus decided to keep the current experimental setup to allow for the widest possible substrate scope to be used in the radiochemistry apparatus. In summary, the radioactive gas handling system delivered [^11^C]CO into the reaction vessel within 7 min after end of bombardment (EOB) with a radiochemical yield (RCY) of 71% based on cyclotron produced [^11^C]CO_2_ (Table 1, entry 2).

### 3.1 Purification and formulation system

Radiopharmaceutical purification was performed using semipreparative HPLC with the effluent monitored with ultraviolet and radiation detectors, respectively. A useful feature of the radiochemistry system is the HPLC column scanner, which permits monitoring of radioactivity inside the column during the separation and thus increases confidence in collecting the desired product fraction from the column effluent. A standard protocol for SPE, formulation and sterile filtration was adopted. In short, the fraction was collected into a prefilled vial with sterile water and then pushed through an SPE cartridge to collect the radiopharmaceutical product. Following a rinse with sterile water, the product was next eluted with ethanol (1.5 ml) into a prefilled vial with saline (5 ml) before it was passed through a sterile filter into a sterile product vial, also prefilled with saline (8.5 ml) to provide the end radiopharmaceutical product in a sterile solution containing less than 10% ethanol. After each synthesis, the system is automatically cleaned using a validated cleaning procedure.

### 3.2 Process evaluation—Production of [^11^C]AZ13198083

**SCHEME 1:**
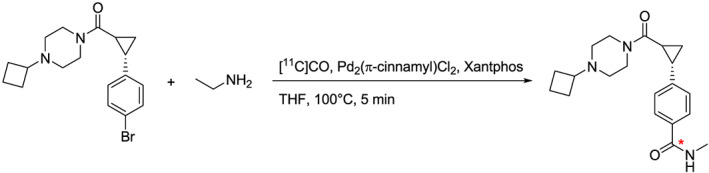
Synthesis of [^11^C]AZ13198083

To evaluate the efficiency of the radiochemical synthesis apparatus, a process evaluation was performed, which composed the production of [^11^C]AZ13198083, a radioligand for imaging the histamine type‐3 receptor (Scheme 1).^17^ Three consecutive batches were produced under “full” cyclotron irradiation conditions (55 μA, 30 min), and each of the batches were subjected to most of the relevant quality control (QC) tests performed prior to release of a GMP manufactured product (Table 2). Gratifyingly, the overall process yielded 8540 ± 1400 MBq of [^11^C]AZ13198083, and each of the batches fulfilled all specifications that were preset for the production (eg, radiochemical purity, pH, and residual solvents). Though tests for sterility and bacterial endotoxins were outside the scope of the current study, our experience is that it is rather straightforward to manufacture sterile and endotoxin free PET radiopharmaceuticals with a suitable cleaning routine. In this context, it is important to also note that a fully automated cleaning protocol allows for the unit to be used to deliver a new batch within 2.5 h after completed production without an unnecessary radiation burden on the operator.

**TABLE 2:** Batch results from the production of [^11^C]AZ13198083

Test	Specification	Batch 1	Batch 2	Batch 3
Radioactivity	N.A.	7050 MBq	8730 MBq	9830 MBq
pH	4.5–8.0	7.0	7.5	7.5
Product identification	R_t_ Radiopeak–R_t_ UV = 0.3–0.5 min	0.35 min	0.33 min	0.38 min
Radiochemical purity	Not less than 95%	>99%	>99%	>99%
Molar activity	N.A.	38.1 GBq/μmol	54.6 GBq/μmol	108.7 GBq/μmol
Filter integrity	Not less than 3.5 bar	3.6 bar	3.7 bar	4.1 bar
Residual acetone	Not more than 5000 ppm	2652 ppm	2097 ppm	2978 ppm
Residual acetonitrile	Not more than 410 ppm	8.9 ppm	Not detected	Not detected
Ethanol content	Not more than 10%	3.6%	3.4%	3.7%

The efficiency of ^11^C‐aminocarbonylation reactions can, in our experience, be improved by dissolving all the components of the reaction mixture (aryl halide, Palladium source, and ligand) in THF (~500 μl) and then purging the solvent to dryness under nitrogen gas flow (~50 ml/min) prior to adding a solution of the appropriate amine shortly before starting the radiopharmaceutical production. Though one may speculate that the oxidative addition product is formed during this process, we have no experimental support for this. Nevertheless, this “premixing” of Pd, ligand, and aryl halide was successfully adopted in the current study.

## 4 CONCLUSION

A fully automated radiochemical synthesis apparatus that enables use of the versatile synthon [^11^C]carbon monoxide as the labeling precursor has been developed. The apparatus is setup for manufacturing according to GMP and comprises all relevant parts of a radiopharmaceutical production, including radiochemistry, purification, formulation, and sterile filtration. During process evaluation, the apparatus was successfully applied to the production of the histamine type‐3 receptor radioligand [^11^C]AZ13198083 in high RCY and fulfilling all set QC specifications. The development of this apparatus paves the way for future applications of [^11^C]carbon monoxide radiochemistry to the production of ^11^C‐labeled drugs and radioligands in a GMP compliant fashion for PET imaging in human subjects.

## 5 EXPERIMENTAL

### 5.1 General experimental information

Unless otherwise stated, all reagents and solvents were obtained from Sigma‐Aldrich (Sweden) and used without further purification. No‐carrier‐added [^11^C]CO_2_ production was performed using a GEMS PETtrace cyclotron (GE, Uppsala, Sweden). The ^14^N(p, α)^11^C reaction was employed in a pressurized gas target containing nitrogen (nitrogen 6.0) and 1% oxygen (oxygen 4.8) by bombardment with a proton beam (16.5 MeV). HPLC was performed using a Hitachi L‐6200 gradient pump and a Hitachi L‐4000 variable wavelength UV‐detector in a series with a Bioscan β^+^‐flow detector. Analytical HPLC was performed using a Hitachi L‐6200 gradient pump and a Hitachi L‐4000 variable wavelength UV‐detector in series with a Bioscan β^+^‐flow detector. The reverse phase column (Agilent Eclipse XDB‐C18, 5 μm, 4.6 × 150 mm) was eluted with a gradient between acetonitrile (A) and 100mM HCO_2_NH_4_ (B). The gradient was linear between 10% and 90% (B) over 5 min at a flow rate of 3 ml/min. Identification of all radioactive products was confirmed by co‐elution with the corresponding nonradioactive compound.

### 5.2 General procedure for ^11^C‐carbonylation at low pressure

The flowchart of the radiochemistry system is shown in Figure 1A,B. At the EOB, the target content was delivered to the system, where [^11^C]CO_2_ was trapped on a Porapak Q column (~650 mg packed in a 1/4″ SS tube, mesh 80/100, Alltech) immersed in liquid nitrogen. The Porapak trap was first flushed with Helium at a flowrate of 400 ml/min for 90 s while still immersed in liquid nitrogen and then flushed during thawing with heated compressed air (flowrate 50 ml/min for 40 s) before [^11^C]CO_2_ was released to the Mo column for reduction. [^11^C]CO_2_ was reduced online to [^11^C]CO using a preheated oven (850°C) quartz glass column (6 × 4 × 220 mm: o.d. × i.d. × length) charged with Mo powder (1.61 g, <150 μm, 99.99% trace metals basis, Sigma Aldrich). Unreacted [^11^C]CO_2_ was subsequently removed by a sodium hydroxide‐coated silica (Ascarite II, 20–30 mesh) trap (Benchmark column 10 mm 100 mm 2xF, 006BCC‐10‐10‐FF) after which the [^11^C]CO was concentrated on a stainless steel trap (1/16″) filled with silica gel (110 mg, 60 Å, 60–100 mesh), immersed in liquid nitrogen. After completed entrapment, the trap was heated to release the [^11^C]CO through a capillary tubing (PEEK, 0.25 mm i.d.) into a closed vial (3.5‐ml glass vial, Chromacol) prefilled with ^11^C‐aminocarbonylation reagents. The resulting solution was then allowed to react at the desired time and temperature. After completed reaction, the crude reaction mixture was diluted with mobile phase and transferred in two portions into an HPLC loop (5 ml, Rheodyne) using a 2.5 ml syringe pump. The solution was then injected onto the HPLC column with an injection valve (Rheodyne, P/N PD700‐113) for purification. The HPLC column effluent was monitored with an ultraviolet detector (Knauer smartline UV detector 200) in series with a radiation detector (LND Inc, 716 gamma detector). In addition, the system was equipped with a column scanner, which consists of a radiodetector moving at low speed (15 mm/s) along the HPLC column, to permit the operator to follow the movement of radioactivity inside the column during the separation. The purified product was then collected from the HPLC column into a prefilled vial with sterile water and then pushed through an SPE cartridge to collect the radiopharmaceutical product. Following a rinse with sterile water, the product was next eluted with ethanol (1.5 ml) into a prefilled vial with saline (5 ml) before it was passed through a sterile filter into a sterile product vial, also prefilled with saline (8.5 ml) to provide the end radiopharmaceutical product in a sterile solution containing less than 10% ethanol. Thirteen two‐port, two‐way valves (V1–V13, SMC, P/N VDW14JAXB) as well as 38 three‐port, two‐way valves (V14–V52, Bürkert, P/N Type 6624‐00241432) were used to direct liquid and gas flow throughout the production procedure. The entire synthesis process was controlled and monitored by an in‐house developed software (NI Labview). The movement of radioactivity in the system was monitored with a series of radiation detectors (LND INC, USA).

### 5.3 Synthesis of [^11^C]AZ13198083

Pd_2_[π‐cinnamyl]Cl_2_ (2.2 mg; 1 eq), xantphos (5.0 mg; 2 eq), ((2S)‐2‐(4‐bromophenyl)cyclopropyl)(4‐cyclobutylpiperazin‐1‐yl)methanone (3.3 μl) were dissolved in anhydrous THF (700 μl), and the resulting mixture was flushed with N_2_ until the THF was fully evaporated (20 min). A solution of ethylamine in THF (2M, 600 μl) was added, and the reaction vial was subjected to a leak check prior to its installation into the radiochemistry system. The ^11^C‐carbonylation reaction was performed during 5 min at 100°C, after which the crude product was purified using a semipreparative HPLC column (ACE C‐18, 5 μm, 10 × 250 mm; Advanced Chromatography Technologies Ltd; Aberdeen, UK) eluted with an isocratic mobile phase (30% MeCN in HCO_2_NH_4_ (0.1M)) at 6 ml/min. The eluate was monitored for absorbance (λ = 254 nm) and for radioactivity. [^11^C]AZ13198083 (t_R_ = 8 min) was collected and diluted with water (~50 ml) and then concentrated on a C‐18 Sep‐Pak (Waters, Sweden). The Sep‐Pak was washed with water (~8 ml) prior to elution with ethanol (99%, 1.5 ml) into saline (5 ml). The formulation was finally filtered through a sterile filter (0.22 μm, Millipore, Millex®GV) into a prefilled vial containing an additional portion of saline (8.5 ml) to yield the final product in a sterile injection vial and in a solution suitable for intravenous injection.
